# Estimation of Health-Related Physical Fitness (HRPF) Levels of the General Public Using Artificial Neural Network with the National Fitness Award (NFA) Datasets

**DOI:** 10.3390/ijerph181910391

**Published:** 2021-10-02

**Authors:** Seung-Hun Lee, Hyeon-Seong Ju, Sang-Hun Lee, Sung-Woo Kim, Hun-Young Park, Seung-Wan Kang, Young-Eun Song, Kiwon Lim, Hoeryong Jung

**Affiliations:** 1 Division of Mechanical and Aerospace Engineering, Konkuk University, 120 Neungdong-ro, Gwangjin-gu, Seoul 05029, Korea; erioer95@konkuk.ac.kr (S.-H.L.); hopeju76@konkuk.ac.kr (H.-S.J.); lshkn2000@konkuk.ac.kr (S.-H.L.); skang103@konkuk.ac.kr (S.-W.K.); 2 Physical Activity and Performance Institute, Konkuk University, 120 Neungdong-ro, Gwangjin-gu, Seoul 05029, Korea; kswrha@konkuk.ac.kr (S.-W.K.); parkhy1980@konkuk.ac.kr (H.-Y.P.); exercise@konkuk.ac.kr (K.L.); 3 Department of Sports Medicine and Science, Graduate School, Konkuk University, 120 Neungdong-ro, Gwangjin-gu, Seoul 05029, Korea; 4 Department of Electrical Engineering, Hoseo University, 20 Hoseo-ro 79 beon-gil, Baebang-eup, Asan-si 31499, Korea; tdsong@hoseo.edu; 5 Department of Physical Education, Konkuk University, 120 Neungdong-ro, Gwangjin-gu, Seoul 05029, Korea

**Keywords:** smart fitness, artificial neural network, health-related physical fitness level estimation

## Abstract

Estimation of health-related physical fitness (HRPF) levels of individuals is indispensable for providing personalized training programs in smart fitness services. In this study, we propose an artificial neural network (ANN)-based estimation model to predict HRPF levels of the general public using simple affordable physical information. The model is designed to use seven inputs of personal physical information, including age, gender, height, weight, percent body fat, waist circumference, and body mass index (BMI), to estimate levels of muscle strength, flexibility, maximum rate of oxygen consumption (VO_2max_), and muscular endurance. HRPF data (197,719 sets) gathered from the National Fitness Award dataset are used for training (70%) and validation (30%) of the model. In-depth analysis of the model’s estimation accuracy is conducted to derive optimal estimation accuracy. This included input/output correlation, hidden layer structures, data standardization, and outlier removals. The performance of the model is evaluated by comparing the estimation accuracy with that of a multiple linear regression (MLR) model. The results demonstrate that the proposed model achieved up to 10.06% and 30.53% improvement in terms of R^2^ and SEE, respectively, compared to the MLR model and provides reliable estimation of HRPF levels acceptable to smart fitness applications.

## 1. Introduction

Smart fitness that provides intelligent and personalized training management services without the intervention of a human trainer has attracted great attention in recent years [1,2,3,4]. It provides high-quality and customized nutrition and workout management at low cost by adopting recent cutting-edge technologies, including artificial intelligence, big data, and internet of things sensors [2]. Recently, smart fitness has grown its application area through smart phone applications and is gradually getting into the offline fitness clubs. However, several technical issues need to be resolved to enhance its service quality and effectiveness. The measurement of the health-related physical fitness (HRPF) level of an individual user, which is required for selecting tailored training programs, is challenging in smart fitness development. Current physical fitness assessment protocols, such as one repetition maximum (1RM) and the maximum rate of oxygen consumption (VO_2max_), may require complicated procedures accompanied by expensive equipment and experts, causing inconvenience and increase in the cost [5,6,7]. Due to insufficient methods for a convenient measurement of personal HRPF levels by the users themselves, the current smart fitness applications inevitably involve the intervention of human trainers to select customized workout programs according to trainer suggestions or user preferences [2,8,9,10]. Several previous studies have aimed at developing methods for estimating an individual’s HRPF level by using simple attainable information, such as height, weight, body mass index (BMI), and percent body fat.

Multiple linear regression (MLR) assuming a linear relation between input and output variables has been used to predict HRPF levels in the field of sports sciences. MLR-based prediction models have shown reliable estimation results with high R^2^ values [11,12]. However, MLR models have the critical limitation that they cannot represent nonlinearity in the target system. Therefore, machine learning techniques that can represent non-linear characteristics, including the ordinary forest algorithm and support vector machine (SVM), have been implemented to improve estimation accuracy of the prediction model. Mahajan et al. proposed an ordinary forest algorithm to predict divers’ competition scores using their physical fitness levels [13]. The proposed model, which was trained by previous competition records and the corresponding physical fitness levels of 231 divers, presented 16.9% improvement in estimation accuracy over the previous linear regression model. Akay et al. proposed an SVM-based estimation model to predict the strength of hamstrings and quadriceps using athletes’ body information [14]. The estimation model was trained with the data acquired from 75 athletes in Gazi University and showed superior prediction accuracies (R^2^ = 0.884). Taha et al. conducted a study to estimate the expertise of 50 young archers based on their physical fitness level using the k-nearest neighbors (k-NN) and SVM algorithms [15,16]. They identified that core muscle strength and vertical jumps are important factors in predicting archers’ potential. They also confirmed that the weighted k-NN algorithm produced results that were 2.5% better than the linear regression model.

Artificial neural networks (ANNs) can better predict complicated features involving nonlinearities than a regression model and all other machine learning algorithms. Several studies have developed superior estimation models for measuring the physical fitness level of humans using an ANN framework. Ruiz et al. developed an ANN-based VO_2max_ estimation model using test results of a 20-m shuttle run from 193 subjects [17]. The proposed ANN model improved the standard errors of estimation (SEE) up to 1.43 (mL/kg min) compared with the Le’ger’s equation. Kupusinac et al. proposed an ANN-based percent body fat estimation model using age, gender, and BMI [18]. The model was trained with the data gathered from 4178 subjects and recorded up to 3.12% higher prediction accuracy than the MLR model. Nicholas et al. performed a study to compare the estimation accuracy of an ANN model with an MLR model in manual arm strength (MAS) prediction [11]. The MAS dataset collected from 95 healthy female participants was used as training and validation data, and the results showed that the prediction accuracy of the ANN model was 11.8% higher than that of the MLR model.

Although previous studies verified that machine learning models (MLR, SVM, and ANN) can estimate individual physical fitness levels using simple physical information with acceptable accuracies, these models have limitations when applied to the general public because the datasets to train and validate the models were gathered from a particular group of people (athletes) [13,14,15,16]. In addition to existing studies that require strict procedures with guidance from experts in the data acquisition, other studies require complicated equipment for measurement, thereby posing additional limitations when applied to ordinary people [19].

In this study, we propose an ANN-based estimation model to predict the HRPF level of the general public using the subject’s attainable physical information as inputs for smart fitness development. The public dataset called the ‘National Fitness Award’ (NFA) from the Korea Sports Promotion Foundation (KSPO) was used to train the proposed ANN-based estimation model to represent the HRPF characteristics of the general public in South Korea. The proposed model was developed to estimate four HRPF indices, namely flexibility, muscular strength, cardiorespiratory endurance, and muscular endurance, using physical information as inputs for easy and convenient determination of the HRPF level. In addition, we investigated the effects of several techniques, including data refinement, normalization, and correlation analysis of input/output variables, on the estimation accuracy of the ANN model to derive the optimized ANN estimation model. The key contributions of this study are as follows.

1. This paper proposes the ANN-based estimation model to predict the HRPF level of the general public using NFA datasets. The proposed model aims to improve the estimation accuracy of the previous MLR-based estimation model by additionally adopting a non-linear feature in the estimation model. ANN structures including layer and node configurations, non-linear activation function, and training/validation methods for the estimation model are presented.
2. This paper derives the optimal ANN model that maximizes the estimation accuracy through in-depth analysis on effects of four techniques including input-output correlation, hidden layer structures, input data standardization, and outlier removal to the estimation accuracy. Contributions of each technique to the final accuracy results are quantitatively evaluated in terms of R^2^ and SEE values and the optimal ANN model is proposed based on the results of the analysis.
3. Finally, this paper proves the superior performance of the ANN model by comparing the estimation accuracy of the ANN model with not only that of the previous MLR model but also that of the representative machine learning models including K-NN, random forest, and support vector machine (SVM). It demonstrates practical usefulness of the proposed model in smart fitness applications.

## 2. Materials and Methods

### 2.1. Dataset

The NFA dataset, which is established by the KSPO for improving physical fitness and health of the South Korean general public, was used to train the ANN-based estimation model for HRPF level prediction. The NFA dataset consists of HRPF level data, which is measured under stringent measurement protocols in 75 sites distributed throughout South Korea, and physical information of the corresponding subjects. The participants for NFA datasets were selected among healthy Korean volunteers older than 10 years. All volunteers undertook the physical activity readiness questionnaire (PAR-Q) to identify whether he/she was eligible for HRPF measurements. Only the volunteers who were determined to be able to take the measurement were approved to participate in the data gathering. The KSPO gathers new HRPF data from more than 100,000 participants every year and is easily accessible through the KSPO website [20]. Initially, we downloaded 456,663 sets of data, collected between 2015 and 2019, from the website, out of which we secured 197,719 sets of data consisting of 87,188 datasets measured from male participants and 110,531 datasets measured from female participants; we excluded 258,914 sets of data that contained missing values from the initial download. The excluded datasets were distributed randomly and uniformly over the entire age range and gender, so there was no bias caused by data exclusion. This study was conducted according to the guidelines of the Declaration of Helsinki and approved by the Institutional Review Board of Konkuk University (7001355-202101-E-132). Table 1 summarizes the NFA dataset used for training and validation of the model. We used 70% and 30% of the data as the training and test/validation datasets, respectively.

The physical information of each participant was measured under the strict measurement protocols [21]. Height was measured using a stadiometer (Seca, Seca Corporation, Columbia, MD, United States) to the nearest 0.1 cm. Weight and percent body fat were measured using bioelectrical impedance analysis equipment (Inbody 720, Inbody, Korea). Waist circumference was measured using a tapeline at the middle of the lowest position of the ribs and the highest position of the pelvis. BMI was calculated by dividing body weight (kg) by height squared (m^2^). The HRPF levels in the NFA are evaluated against four fitness indices, namely flexibility, muscular strength, cardiorespiratory endurance (VO_2max_), and muscular endurance, and measured as follows:

*Flexibility:* This is measured by the sit and reach test. The participants sit against a wall with knees fully extended and maintain a distance of <5 cm between their feet. After placing the hands on point zero of the measuring ruler, the participants reach them out as far forward as possible, lining up with the ruler. The distance between the starting point to the tip of the finger is measured by the ruler.

*Muscle strength:* This is measured by the grip strength test. The participants align their second knuckle of their fingers to the grip of the dynamometer. Then, they squeeze the dynamometer for 5 s with the arm straight while maintaining the torso and arm held at an angle of 15°. They perform this procedure two times alternating between the left and right hands. Accordingly, the highest value among the four measurements is recorded.

*Cardiorespiratory endurance (VO_2max_):* This is measured using a 20-m shuttle run test. The participants, standing on the starting line, start running when the beep sounds. When they arrive at the 20 m mark, they wait and return to the start line after the next beep. The interval between the beeps will gradually decrease with every repetition from 8 s. One run of 20 m counts as one repetition, and participants will repeat the same process until there are two failures to arrive at the 20-m mark distance before the beep sounds. The VO_2max_ is calculated by the regression equation developed by the Korea Institute of Sports Science (KISS) [22].

*Muscular Endurance:* This is assessed by measuring the number of sit-ups. The participants lie on a mat with knees bent at 90° and their feet placed on the measuring instrument. Their arms are overlapped in an X-shape above the chest and holding onto their shoulders. The participants raise their upper body back and forth for 1 min and both elbows must touch the thighs to make it count as 1 repetition.

### 2.2. ANN-Based HRPF Level Estimation Model

**Figure 1:**
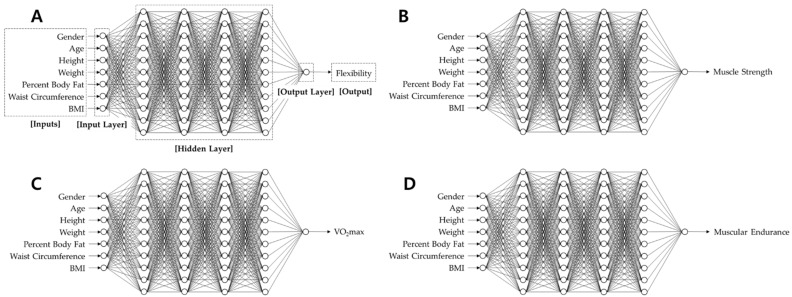
Overall architecture of the ANN-based HRPF level estimation model. (**A**) Architecture of the ANN model for predicting flexibility with descriptions, (**B**) Architecture of the ANN model for predicting muscle strength, (**C**) Architecture of the ANN model for predicting VO_2max_, (**D**) Architecture of the ANN model for predicting muscular endurance.

Figure 1 shows the overall architecture of the ANN-based estimation model that predicts the personal HRPF level of participants by using their physical information as inputs. The ANN model consists of three layers: input, hidden, and output. The input layer consists of a total of seven nodes, each of which receives one of seven elements of the physical information: age, gender, height, weight, percent body fat, waist circumference, and BMI. The hidden layer comprises multiple layers and nodes, and the optimal hidden layer structure is derived by comparing the accuracy of several layer configurations (the number of layers and nodes). The exponential linear unit (ELU) function is used as an activation function for nonlinear estimation. The output layer comprises a single node, and it yields an estimated HRPF level from the given inputs. In this study, four ANN models with identical layer structures were used to estimate the four HRPF levels of muscle strength, muscular endurance, cardiorespiratory endurance, and flexibility.

The ANN-based estimation model was trained with the refined NFA dataset consisting of 138,281 sets of input/output pairs, and 5-fold cross validation was used in the training. The mean square error (MSE) was used for calculating the loss function in the training, and Adam (learning rate: 0.02, beta1 = 0.9, beta2 = 0.99) was used as an optimizer. Each hidden layer contains the normal initializer and batch normalization [23,24]. R^2^ and SEE were used as performance indices for evaluating the accuracy of the trained estimation model. Equation (1) represents the expression for the calculation of SEE:

(1) $$SEE=\frac{\sum_{i=0}^{N}{\left(y_{i}-{\hat{y}}_{i}\right)}^{2}}{N-2}$$

where $y_{i}$, ${\hat{y}}_{i}$, and N are the measured value, estimated value, and number of test samples, respectively.

### 2.3. Optimizations for Enhancing Estimation Accuracy of ANN Model

The correlation between the input and output variables, hidden layer structures, and the quality of the training data can affect the accuracy of the ANN-based estimation model. In this study, we analyzed the effect of these factors on the estimation accuracy to derive the optimal estimation model.

#### 2.3.1. Correlation Analysis between Input and Output Variables

The ANN-based estimation model receives seven different elements of physical information as inputs, which may differ in the correlation between each input variable and target HRPF index. Input variables that are less correlated with the target HRPF index may have a negative effect on the estimation accuracy. Therefore, the estimation accuracy can be improved by selecting highly correlated physical information as inputs to the ANN-based estimation model [25,26,27,28]. The correlation between the seven elements of physical information (input variables) and four HRPF indices (output variables) is quantitatively evaluated using the Boruta algorithm to determine the optimal input configurations for each HRPF index [29].

#### 2.3.2. Comparison of Hidden Layer Structures

The number of parameters for estimating the output in ANN models is determined by the structure of the hidden layer. Overfitting and underfitting problems, which may degrade the estimation accuracy, can arise from too many or too few parameters in the hidden layer. By varying the number of layers and nodes from 1 and 7 to 4 and 28 respectively, we identified the optimal hidden layer structure with maximum estimation accuracy among these 14 configurations.

#### 2.3.3. Standardization of Input Data

Discrepancies in the range of input values (minimum, maximum, average, and standard deviation) can produce outputs biased to the specific inputs whose value ranges are larger than the others. Z-score standardization, expressed by Equation (2), is applied to input values of the seven elements of physical information in the training dataset to avoid this output bias problem. Average and standard deviation of all input values are set to 0 and 1, respectively, through the standardization process.

(2) $${\hat{x}}_{i}=\frac{x_{i}-\mu }{\sigma }$$

where, $x_{i}$, ${\hat{x}}_{i}$ denotes the values of each input variable before and after the standardization, and μ, σ are the average and standard deviation of the corresponding input variable, respectively.

#### 2.3.4. Outlier Removal

An outlier is defined as a data sample that differs significantly from other samples. The outliers included in the training dataset can negatively affect the estimation accuracy of the HRPF level of the general public. This is because outliers convey the characteristics of extreme cases, which is rare among the general public. The outliers are removed by using the interquartile range (IQR) and standard deviation, and the estimation accuracies of these methods are compared to identify the best outlier removal method. In the first case, the outlier is defined using IQR values, which is calculated by $IQR=Q_{3}-Q_{1}$, where $Q_{1}$ and $Q_{3}$ denote the 25th and 75th percentiles, respectively, as shown in Equation (3). The second case determines the outliers as samples whose values lie outside ±3σ, as shown in Equations (4).

(3) $${\bar{x}}_{i}<Q_{1}-1.5\times IQR\;or\;{\bar{x}}_{i}>Q_{3}+1.5\times IQR$$

(4) $${\bar{x}}_{i}<\mu -3\sigma \;or\;{\bar{x}}_{i}>\mu +3\sigma$$

where ${\bar{x}}_{i}$ denotes the outlier in the training datasets. 

## 3. Results

### 3.1. Input/Output Correlation Analysis

**Figure 2:**
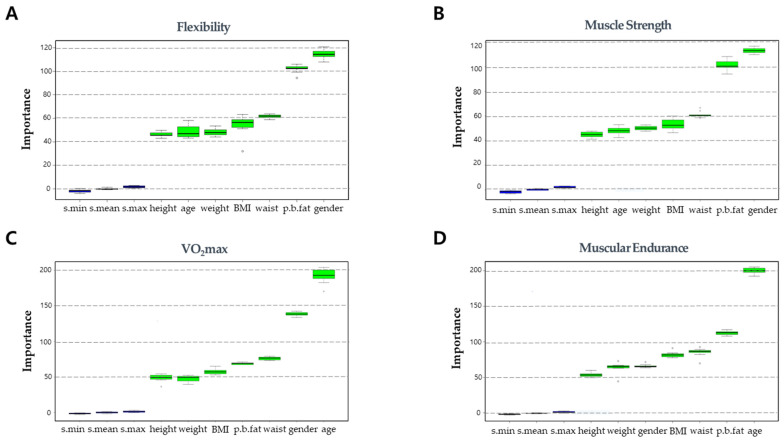
Importance plot demonstrating degree of correlation between input and output variables. S.min, S.max, and S.mean denote the threshold values. (**A**) Importance plot for flexibility, (**B**) Importance plot for muscle strength, (**C**) Importance plot for VO_2max_, (**D**) Importance plot for muscular endurance.

Figure 2 presents the importance plot of the Boruta algorithm, which quantitatively demonstrates the degree of correlation between the input and output variables with importance value. The input variables whose importance value is higher than the threshold, which is denoted by shadow values (min, max, mean) in the graph, can be regarded as having a strong correlation with the corresponding output variable. As shown in Figure 2, the importance values of all seven input variables (physical information) exceed the threshold for all output variables, indicating a strong correlation between the input variables and the HRPF indices. Thus, these input variables can be used for the ANN-based HRPF estimation model. The flexibility and muscular strength showed identical correlation patterns with respect to the importance ranking of input variables. The rank of all the input variables were identical for these two HRPF indices. The cardiorespiratory endurance (VO_2max_) and muscular endurance also showed similar correlation patterns to one another. The input variables presenting the highest and lowest importance value were identical, i.e., age and height, respectively, for these two HRPF indices, and the other variables exhibited little difference. This result offers reasonable evidence for using personal physical information for estimating personal HRPF levels, and the seven input variables are appropriate for the ANN-based estimation model.

### 3.2. Effect of Hidden Layer Structure

Table 2 presents the estimation accuracy of the ANN-based HRPF estimation model for 16 combinations of the hidden layer structures. The accuracy was evaluated using the R^2^ and SEE values. Open-source predictive data analysis library (scikit-learn, version 0.23.2) in python 3.8.5 was used to compute R^2^ and SEE values. The variation of R^2^ and SEE according to the hidden layer structures was not significant. The hidden layer structure with 3 layers and 28 nodes for flexibility, 4 layers and 14 nodes for muscle strength, 1 layer and 7 nodes for VO_2max_, 4 layers and 21 nodes for muscular endurance was selected for the estimation model of each HRPF level as it showed the best performance in R^2^ and SEE. R^2^ values of the models were 0.1844, 0.7476, 0.7394, and 0.5624 for flexibility, muscle strength, VO_2max__,_ and muscular endurance, respectively. For SEE, the model resulted in 8.3540, 5.1381, 3.3305, and 10.5903 for flexibility, muscle strength, VO_2max,_ and muscular endurance respectively.

### 3.3. Effect of Input Data Standardization

Table 3 demonstrates the effect of input data standardization on the estimation accuracy. The table shows R^2^ and SEE values of the model with and without input data standardization. The ANN models that have identical hidden layer structures (Section 3.2) derived above were used for the comparison. The results showed a little improvement in the estimation accuracy through the input data standardization. R^2^ values were improved up to 2.4750% and SEE values were improved up to 3.5100% for VO_2max_ through the input data standardization.

### 3.4. Effect of Outlier Removal

The outliers contained in the training dataset were removed using IQR and 3σ as described in Section 2.3.4. Table 4 presents a comparison of the accuracies of the model with and without implementing the two outlier removal methods to demonstrate the effect of the outlier removal on the estimation accuracy. The outlier removal was performed using the dataset with input data standardization. The ANN models used for the comparison had identical hidden layer structures. The number of data removed through the outlier removal were 12,250 and 6532 in IQR and 3σ respectively. Compared to the model without outlier removal, the R^2^ values were improved up to 1.2329% for muscle strength and SEE were improved up to 6.5503% for VO_2max_ through IQR outlier removal.

### 3.5. Performance Comparison with the MLR Model

**Figure 3:**
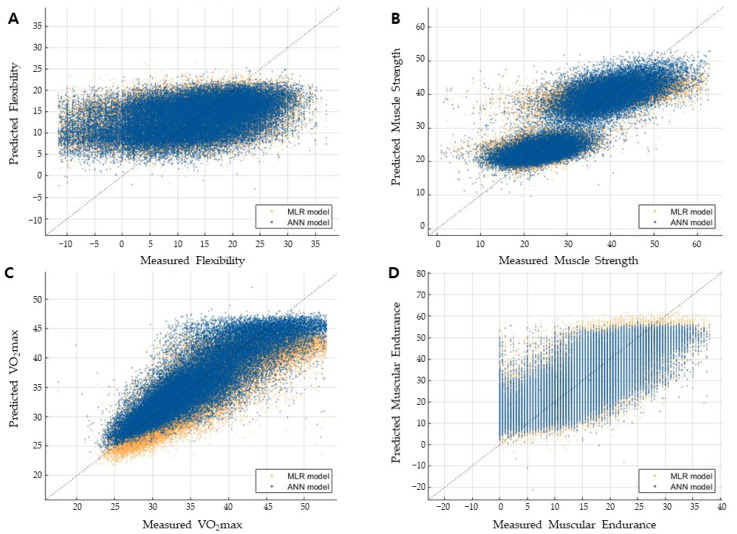
Comparison of scatter plot between the ANN and the MLR estimation model for four HRPF indices. (**A**) Scatter plot for flexibility, (**B**) Scatter plot for muscular strength, (**C**) Scatter plot for VO_2max_, (**D**) Scatter plot for muscular endurance.

The performance of the ANN-based estimation model, consisting of the optimal hidden layer structure, input data standardization, and outlier removal using IQR, was compared with the MLR-based estimation model [21] and other machine learning models including K-Nearest Neighbor (K-NN), random forest, and support vector machine (SVM), using the identical NFA dataset as presented in Table 5. The results demonstrates that the proposed ANN model shows superior performance compared to the other machine learning models and MLR model for three HRPF indices except flexibility. The proposed ANN model presented the best performance in terms of both R^2^ and SEE values for estimation of muscle strength and VO_2max_. R^2^ was improved ranging from 1.3900% to 6.3675% and 1.1927% to 5.7536% for muscle strength and VO_2max_ respectively. SEE also was improved ranging from 3.6106% to 12.5308% and 7.7653% to 30.5318% for muscle strength and VO_2max_ respectively. For muscular endurance, the SEE value was improved, ranging from 1.0888% to 2.9772%, while R^2^ remained similar to the other machine learning models. The MLR model has shown the worst performance for all four HRPF indices compared to the machine learning models. Figure 3 shows the scatter plot of the ANN model and MLR model. As shown in the figure, the bias and deviations were improved in the ANN model.

## 4. Discussion

There have been various studies conducted to predict the level of physical fitness using machine learning techniques. However, the existing research results difficult to adopt into the smart fitness applications aimed at the general public since the previous studies mainly focused on a specific group such as athletes. In this study, we proposed an ANN-based estimation model that is designed to predict individual HRPF levels of the general public using simple affordable personal physical information. This paper utilizes the public big data called the NFA dataset, which is collected by a public institute of South Korea. In total, 197,719 sets of HRPF data, each of which consists of seven physical variables, and HRPF levels measured by four indices, were used for training and validation of the ANN model. In virtue of huge datasets, the proposed model achieved reliable estimation results for muscle strength (R^2^ = 0.7554), VO_2max_ (R^2^ = 0.7630), and muscular endurance (R^2^ = 0.5707) that can be adopted in the smart fitness application. However, the estimation accuracy of the flexibility was unsatisfactory as it showed quite a low R^2^ value (R^2^ = 0.1700).

This paper conducted in-depth analyses on input/output correlations, hidden layer structures, input data standardization, and outlier removal to identify the effect of these factors on the estimation accuracy, eventually enhancing the performance of the ANN model. In the input/output correlation analysis using the Boruta algorithm, all seven input variables were identified as important variables that can be regarded as having a significant correlation with the levels of the four HRPF indices. This provides reasonable evidence for using personal physical information for estimating personal HRPF levels. The number of layers and nodes in the hidden layer may affect the estimation accuracy of the ANN model because it determines the total number of parameters used in the estimation. Overfitting and underfitting problems can be caused by inadequate hidden layer structures. The maximum deviation of R^2^ was 0.0541 observed in muscular endurance, whereas the deviations of the others were 0.0226, 0.0252, and 0.0050 in flexibility, muscular strength, and VO_2max_, respectively. This result implies that a HRPF level prediction model can be built with a small number of weights, while an excessive number of weights can reduce the efficiency and accuracy of learning. 

The input variables used for training the ANN model have different scales and ranges, and these can produce biased output to specific inputs. The input data standardization was conducted on the training datasets to prevent output bias. R^2^ values of the muscle strength, VO_2max_, and muscle endurance slightly increased through standardization. The SEE of the model trained by standardized input variables showed a reduction up to 3.5100% for VO_2max_. This result demonstrates that the performance of the ANN-based estimation model can be improved by minimizing the output bias through input data standardization. The NFA dataset used in training the model contains outliers that can lower the prediction performance. The outlier removal using IQR shows the maximum improvement for muscle strength and VO_2max_, while outlier removal with 3σ shows the best performance for the flexibility and muscular endurance, respectively. However, when too much data were removed, data close to the mean value were removed in addition, lowering the estimation accuracy and SEE value in the overall performance evaluation.

The R^2^ and SEE values from the ANN model showed a distinct improvement over the previous MLR model for all indices. The ANN-based estimation model resulted in 10.0588%, 6.3675%, 5.7536%, and 2.4882% improvements in the R^2^ for flexibility, muscle strength, VO_2max_, and muscular endurance, respectively. The SEE was improved by 10.5645%, 12.5308%, 30.5318%, and 2.9772%, respectively. This result demonstrates that the proposed ANN-based estimation model predicts personal HRPF levels more accurately than the previous MLR-based estimation model. Furthermore, one can infer that a nonlinear relation exists between the physical information and HRPF levels that enables performance improvement in the ANN model. The scatter plot presented in Figure 3 consistently supports the superior performance of the ANN model. The overall bias and variance of the estimated values in the ANN model are smaller than those for the MLR model, which results in a higher R^2^ and lower SEE values. In the accuracy comparison with the other machine learning models including K-NN, Random Forest, and SVM, the proposed ANN models showed superior performance in most of HRPF indices, but the amount of improvement was not substantial and was lower than 5% in terms of R^2^. Since the number of input parameters in the HRPF estimation model, which determines the complexity of the model, is relatively simple compared to the other machine learning problems such as image recognition and natural language processing, selection of the machine learning method may not be critical to the estimation accuracy compared to the other complex problems. Simple machine learning models may provide enough performance. Considering the practical use in smart fitness applications, computational efficiency and the ease of implementation across multiple smart fitness devices can be more important factors in choosing the estimation model. 

The proposed ANN model achieved reliable estimation of the HRPF levels, but there remains a limitation that should be addressed in future research. Although the proposed model predicts muscle strength, VO_2max_, and muscular endurance with acceptable accuracies, the estimation accuracy of the flexibility was too low to be used in practical applications. It is inferred that the input variables used for estimating the flexibility are not sufficient to predict the level of flexibility. Hence, additional research is required to improve the estimation accuracy of the flexibility by exploring other variables that have strong correlation with the flexibility.

## 5. Conclusions

In this study, we proposed an ANN-based estimation model to predict the HRPF levels of the general public using the NFA dataset. The estimation accuracy of the ANN model was analyzed in terms of input/output correlation, hidden layer structures, input data standardization, and outlier removal to derive optimal performance of the model. The estimation accuracies of the proposed model were sufficiently reliable for muscle strength, VO_2max_, and muscular endurance to be applicable to smart fitness applications. However, the estimation results of the flexibility were unsatisfactory. This study determined that standardization of data and elimination of outliers are important factors in predicting the HRPF level using the ANN model. In addition, the prediction performance for the four physical fitness levels demonstrates that the performance of the model using ANN was superior to that of the MLR model.

## Figures and Tables

**Table 1:** Summary of the NFA dataset used for training and validation of the ANN model. In total, 197,719 sets (male: 87,188 sets, female: 110,531 sets) of data were used.

Data Type	Index	Max		Min		Mean (SD)	
		Male	Female	Male	Female	Male	Female
Physical Information	Age (year)	64.0	64.0	19.0	19.0	34.6 (14.6)	42.1 (14.7)
	Height (cm)	205.5	196.3	132.9	119.5	172.8 (6.3)	159.2 (5.7)
	Weight (kg)	160.0	143.5	30.4	30.2	73.3 (10.9)	58.2 (8.6)
	Percent Body Fat (%)	63.0	58.6	3.0	2.0	21.0 (6.7)	31.0 (6.5)
	Waist Circumference (cm)	149.0	150.0	50.0	50.2	81.5 (9.4)	81.5 (9.3)
	BMI (kg/m^2^)	48.4	47.1	11.3	11.5	24.5 (3.2)	23.0 (3.3)
Physical Fitness Index	Flexibility (cm)	52.0	40	−30.0	−30.0	9.2 (9.4)	15.0 (8.3)
	Muscule Strength (kg)	78.0	82.2	1.0	1.0	39.8 (7.6)	23.4 (4.7)
	VO_2max_ (mL/kg/min)	67.0	73.8	10.0	13.5	40.5 (5.8)	31.7 (3.9)
	Muscular endurance	100.0	100.0	0.0	0.0	40.3 (14.0)	22.4 (12.8)

**Table 2:** Comparison of estimation accuracy according to hidden layer structures.

# of Layers	# of Nodes	Flexibility		Muscle Strength		VO_2max_		Muscular Endurance	
		R^2^	SEE	R^2^	SEE	R^2^	SEE	R^2^	SEE
1	7	0.1795	8.3789	0.7400	5.2151	**0.7394**	**3.3305**	0.5083	11.2255
	14	0.1798	8.3773	0.7429	5.1855	0.7332	3.3699	0.5483	10.7598
	21	0.1763	8.3954	0.7436	5.1790	0.7280	3.4026	0.5591	10.6302
	28	0.1661	8.4471	0.7404	5.2107	0.7280	3.4027	0.5541	10.6897
2	7	0.1775	8.3892	0.7429	5.1863	0.7373	3.3438	0.5523	10.7116
	14	0.1840	8.3563	0.7425	5.1895	0.7238	3.4288	0.5508	10.7298
	21	0.1838	8.3570	0.7408	5.2070	0.7237	3.4295	0.5553	10.6761
	28	0.1651	8.4522	0.7387	5.2283	0.7140	3.4886	0.5532	10.7010
3	7	0.1731	8.4115	0.7224	5.3888	0.7133	3.4933	0.5493	10.7475
	14	0.1618	8.4688	0.7441	5.1733	0.7315	3.3804	0.5446	10.8032
	21	0.1721	8.4170	0.7443	5.1713	0.7237	3.4294	0.5451	10.7977
	28	**0.1844**	**8.3540**	0.7462	5.1523	0.7387	3.3350	0.5611	10.6060
4	7	0.1764	8.3949	0.7261	5.3522	0.7264	3.4127	0.5441	10.8099
	14	0.1730	8.4122	**0.7476**	**5.1381**	0.7275	3.4058	0.5547	10.6826
	21	0.1751	8.4016	0.7403	5.2118	0.7224	3.4372	**0.5624**	**10.5903**
	28	0.1727	8.4139	0.7394	5.2207	0.6889	3.6387	0.5517	10.7189
Average (±SD)		0.1750 (±0.006)	8.4017 (±0.033)	0.7401 (±0.006)	5.2131 (±0.064)	0.7250 (±0.012)	3.4206 (±0.073)	0.5497 (±0.012)	10.7425 (±0.140)

Note: The values denoted by bold with under bar represents the best cases among the 16 cases respectively.

**Table 3:** Comparison of estimation accuracy according to the input data standardization.

Model	Flexibility		Muscle Strength		VO_2max_		Muscular Endurance	
	R^2^	SEE	R^2^	SEE	R^2^	SEE	R^2^	SEE
Without standardization	0.1844	8.3540	0.7476	5.1381	0.7394	3.3305	0.5624	10.5903
With standardization	0.1832	8.3877	0.7462	5.1527	0.7577	3.2136	0.5735	10.4631

**Table 4:** Comparison of estimation accuracy according to the outlier removal.

Model	# of Data Removed (%)	Flexibility		Muscle Strength		VO_2max_		Muscular Endurance	
		R^2^	SEE	R^2^	SEE	R^2^	SEE	R^2^	SEE
Without	0	0.1832	8.3877	0.7462	5.1527	0.7577	3.2136	0.5735	10.4631
IQR	12,250 (6.19%)	0.1700	7.8868	0.7554	4.9853	0.7630	3.0031	0.5707	10.3421
3σ	6532 (3.30%)	0.1722	8.1054	0.7529	4.9935	3.1083	0.7578	0.5724	10.3508

‘# of data’ means ‘the number of data’.

**Table 5:** Comparison of estimation accuracy between the four machine learning models and the MLR model.

Model	Flexibility		Muscle Strength		VO_2max_		Muscular Endurance	
	R^2^	SEE	R^2^	SEE	R^2^	SEE	R^2^	SEE
MLR model	0.1529	8.7200	0.7073	5.6100	0.7191	3.9200	0.5565	10.6500
SVM model	0.1635	8.4604	0.7426	5.1890	0.7494	3.2659	0.5626	10.5879
K-NN model	0.1764	8.3950	0.7449	5.1653	0.7539	3.2363	0.5735	10.4547
Random forest model	0.1784	8.4181	0.7445	5.1696	0.7523	3.2472	0.5730	10.4607
Proposed ANN model	0.1700	7.8868	0.7554	4.9853	0.7630	3.0031	0.5707	10.3421

Note: The hyperparameters of SVM, K-NN, and random forest were set as the values that showed the best estimation accuracy. In the SVM model, the cost value was set as 0.5 and the polynomial function of degree 3 was used as the kernel function. In the K-NN model, the number of neighbors (K) was set as 47. In the random forest model, n_estimator, max_depth, min_samples_split, and min_samples_leaf was set as 120, 14, 2, and 1 respectively.

## Data Availability

Restrictions apply to the availability of these data. Data was obtained from [KSPO] and are available [from the authors/at https://www.bigdata-culture.kr/bigdata/user/data_market/detail.do?id=c99df919-f2c9-4ceb-999d-82688c028031, aceessed on 29 September 2021] with the permission of [KSPO].
